# Effect of carbon source perturbations on transcriptional regulation of metabolic fluxes in *Saccharomyces cerevisiae*

**DOI:** 10.1186/1752-0509-1-18

**Published:** 2007-03-27

**Authors:** Tunahan Çakır, Betül Kırdar, Z İlsen Önsan, Kutlu Ö Ülgen, Jens Nielsen

**Affiliations:** 1 Boğaziçi University, Department of Chemical Engineering, 34342, Bebek, Istanbul, Turkey; 2 Center for Microbial Biotechnology, Biocentrum-DTU, Technical University of Denmark, DK-2800, Kgs. Lyngby, Denmark

## Abstract

**Background:**

Control effective flux (CEF) of a reaction is the weighted sum of all fluxes through that reaction, derived from elementary flux modes (EFM) of a metabolic network. Change in CEFs under different environmental conditions has earlier been proven to be correlated with the corresponding changes in the transcriptome. Here we use this to investigate the degree of transcriptional regulation of fluxes in the metabolism of *Saccharomyces cerevisiae*. We do this by quantifying correlations between changes in CEFs and changes in transcript levels for shifts in carbon source, i.e. between the fermentative carbon source glucose and nonfermentative carbon sources like ethanol, acetate, and lactate. The CEF analysis is based on a simple stoichiometric model that includes reactions of the central carbon metabolism and the amino acid metabolism.

**Results:**

The effect of the carbon shift on the metabolic fluxes was investigated for both batch and chemostat cultures. For growth on glucose in batch (respiro-fermentative) cultures, EFMs with no by-product formation were removed from the analysis of the CEFs, whereas those including any by-products (ethanol, glycerol, acetate, succinate) were omitted in the analysis of growth on glucose in chemostat (respiratory) cultures. This resulted in improved correlations between CEF changes and transcript levels. A regression correlation coefficient of 0.60 was obtained between CEF changes and gene expression changes in the central carbon metabolism for the analysis of 5 different perturbations. Out of 45 data points there were no more than 6 data points deviating from the correlation. Additionally, up- or down-regulation of at least 75% of the genes were in qualitative agreement with the CEF changes for all perturbations studied.

**Conclusion:**

The analysis indicates that changes in carbon source are associated with a high degree of hierarchical regulation of metabolic fluxes in the central carbon metabolism as the change in fluxes are correlating directly with the change in transcript levels of genes encoding their corresponding enzymes. For amino acid biosynthesis there was, however, not found to exist a similar correlation, and this may point to either post-transcriptional and/or metabolic regulation, or be due to the absence of a direct perturbation on the amino acid pathways in these experiments.

## Background

Metabolic fluxes are functions of metabolite levels, enzyme properties (affinities and specific activities), and the concentrations of enzymes. The latter are controlled at transcriptional, translational and/or post-translational levels, and is therefore referred to as hierarchical regulation [1,2]. In the field of functional genomics, there has been several studies on whether flux regulation is through the expression levels of metabolic genes [3-5], and a common approach is to compare flux levels calculated by flux balance analysis (FBA) or metabolic flux analysis (MFA) with mRNA levels [5-7]. Since many reactions in the metabolic network are not active at the optimum growth conditions determined by FBA, this approach does not enable evaluation of whether there is such a correlation for all genes. There is a similar problem with using MFA, as this approach also only provides information of a limited set of fluxes [3]. Moreover, in FBA the occurrence of alternate optima cannot be excluded causing further uncertainty [8-10]. Additionally, organisms with different flux states may coexist in the same culture. It was previously shown that these approaches do not account for the flexibility of the metabolic network and that the quality of the resultant prediction is greatly improved by the incorporation of flexibility [11]. Elementary flux modes identified by the enumeration of the flux solution space using linear algebra [12] may be a way to provide the missing flexibility information. Thus, weighted sum of fluxes through these elementary modes for each reaction, called control-effective fluxes (CEF), lead to the implicit incorporation of functionality and regulation into metabolic network structures [11,13,14]. CEF changes were previously used for the prediction of transcriptome changes in carbon source shifts for *E. coli *[11] and *S. cerevisiae *[13] metabolisms. Application to erythrocyte enzymopathies was also demonstrated [15].

**Figure 1:**
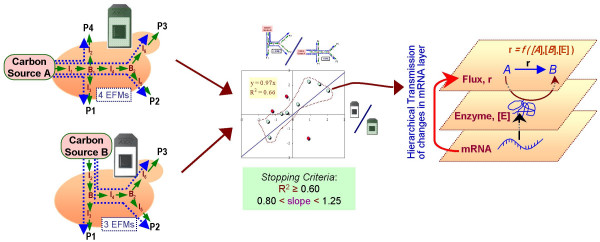
Methodology employed to analyze metabolic flux regulation in response to a shift in carbon source. The change in control-effective fluxes calculated based on determined elementary flux modes (EFMs) is plotted against the change in mRNA levels of the corresponding genes encoding the enzymes catalyzing the reactions. Data points not obeying the correlation are identified by a genetic algorithm approach. Remaining points correspond to hierarchically regulated fluxes.

In this work, the metabolic model for *S. cerevisiae *used in our previous study [13] was improved and extended by the addition of reactions responsible for the amino acid biosynthetic pathways. The resulting metabolic model contains 77 metabolites and 81 reactions which are governed by a total of 137 genes (Additional File 1). Elementary flux modes of this reaction network were calculated for growth on different carbon substrates to determine CEFs. The fold changes of CEFs of reactions in the model in response to perturbations arising from carbon shifts were compared with the fold changes in expression levels of genes encoding the enzymes of the corresponding reactions. The number of fluxes obeying an acceptable correlation was used to evaluate whether the metabolic fluxes are transcriptionally regulated at the studied perturbations. The here-presented methodology is described in Figure 1.

## Results

The set of experimental gene expression data for carbon source perturbations used in this study are summarized in Table 1. If multiple genes correspond to a single reaction, their expression levels were summed up for each condition before the fold change was calculated, and in the following we use the term 'a gene' for both a single gene but also as a representation for several genes involved in a single reaction. The metabolic model consists of central carbon metabolism reactions as described by [13], and was improved by the addition of reactions involved in the synthesis of several amino acids (Additional File 1). Biosynthesis of amino acids that only contribute to a small fraction of the protein in *S. cerevisiae *[16] were directly incorporated into the biomass reaction (r_81a_) rather than including individual reactions responsible for their formation in the model. The metabolic model could therefore be kept at a manageable size. This processing of the model was particularly necessary to avoid combinatorial explosion in the number of elementary flux modes with an increase in the number of considered reactions [17,18]. The stoichiometric coefficients of the reaction leading to biomass formation were calculated on the basis of the biomass composition given by [16].

The number of EFMs calculated for each carbon source is given in Table 2 for the model in Additional File 1, called M81 (based on the number of included reactions), and for a modified version of this model, M57, which only includes central carbon metabolism reactions as in [13]. When the number of EFMs of the two models is compared, an approximately ten-fold increase is observed for M81 compared with M57. Therefore, it may be concluded that the inclusion of amino acid reactions enables better and less-restricted representation of the network flexibility. The coefficients of biomass constituents were calculated also on the basis of another cellular macromolecular composition reported by [19] for *S. cerevisiae *(r_81b_), and this calculation led to noticeable differences in the resultant number of EFMs for the same carbon source (Table 2). However, variations between calculated CEFs for the two different set of EFMs were small and, therefore, the biomass composition does not seem to influence the analysis substantially. In our analysis we used the biomass composition described in [16].

In order to identify active EFMs during growth on glucose, basic information on the yeast metabolism was used. Thus, the following strategy was pursued: For batch experiments (Table 1), where metabolism is respiro-fermentative, EFMs producing any of the byproducts ethanol, glycerol, acetate and succinate were retained since this mode is mainly associated with simultaneous biomass and product formation, whereas those producing only biomass were discarded, reducing the number of EFMs from 136,925 to 127,872. For chemostat experiments, where there is hardly any metabolites being produced [3], only EFMs leading to biomass without co-current metabolite production were considered, This resulted in 9,600 EFMs instead of 136,925. EFMs with only ATP maintenance activity were kept in both cases. This approach was used to test the prediction capabilities of the previous models where all the EFMs had been used without such distinction for comparison with experimental data [11,13], and the present strategy to include only active EFMs into the model was found to enable improved correlations between gene expression changes and changes in CEFs (results not shown). Our approach can be seen as a reduction in the possible flexibility in operation of the metabolic network, but using our approach we indirectly incorporated information on overall regulation of respiratory metabolism in *S. cerevisiae*, i.e. at conditions with fermentative metabolism respiration is to a large extent repressed and at respiratory metabolism there is hardly any substantial metabolite production.

For each case (Table 1), the operation efficiency of each EFM was quantified in terms of its biomass production flux (r_81b_) and ATP production flux for maintenance purposes (r_51_) (see Methods section for details). The resulting score, called the efficiency, was used as a weight for the fluxes passing through the corresponding EFM. As a result, control-effective flux of a reaction represents the sum of all weighted fluxes for this particular reaction that may participate in all the EFMs. Both efficiency and flexibility of the network is thereby reflected in the CEF score. The correlation between fold changes in CEFs and in mRNA levels were then investigated as depicted in Figure 1.

**Figure 2:**
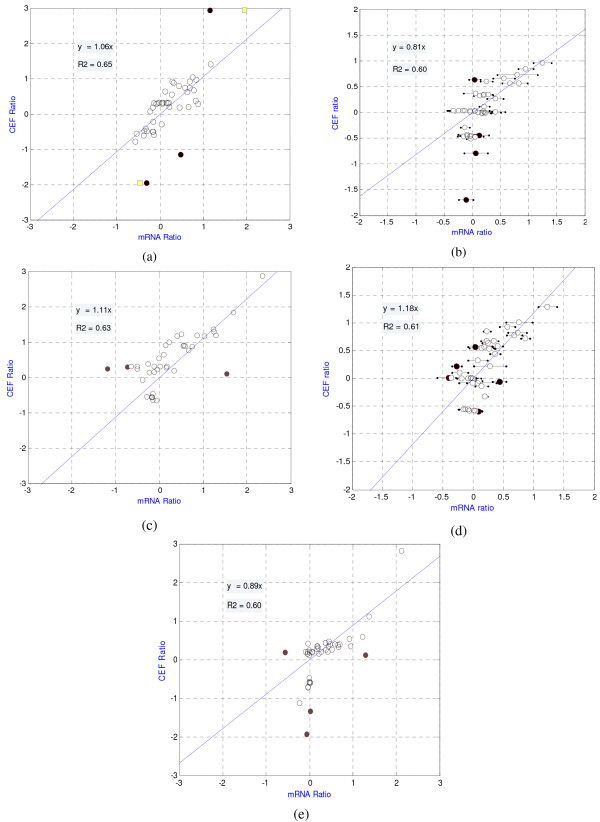
Comparison of the model-based and experimental data-based logarithmic ratios for carbon shifts. The filled circles are the omitted points to reach regression coefficient R^2 ^0.60. (a) shift from glucose to ethanol for batch cultures under respiro-fermentative conditions (data from [20]). Filled squares belong to ratios for pfk12 and fbp1 from [21]. (b) shift from glucose to ethanol for chemostat cultures under respiratory conditions (data from [3]). (c) shift from glucose to acetate for batch cultures under respiro-fermentative conditions (data from [25]). (d) shift from glucose to acetate for chemostat cultures under respiratory conditions (data from [3]). (e) shift from glucose to lactate for batch cultures under respiro-fermentative conditions data from [30]. For experiments with replicate data (b and d), the horizontal lines on the circles show 95% confidence intervals.

**Figure 3:**
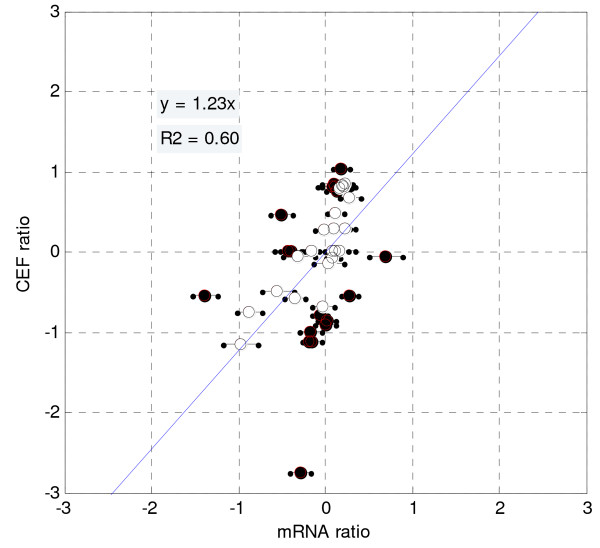
Comparison of the model-based and experimental data-based [36] logarithmic ratios for oxygen source perturbation from aerobic to anaerobic conditions in chemostat cultures. The filled circles are the omitted points to reach the selected threshold value of R^2 ^= 0.60. The horizontal lines on the circles show 95% confidence intervals, calculated from standard deviation of the replicate measurements.

Table 3 summarizes the simulation results for genes belonging to the central carbon metabolism (45 points in total) for each of the cases listed in Table 1. The results include the fraction of genes in first/third quadrants of plots (qualitative agreement), the correlation coefficient, the slope, and the number of omitted genes to reach R^2 ^= 0.60. An acceptable correlation (R^2 ^= 0.60, see methods) with a slope close to unity was possible by omitting at most 6 points for all the studied carbon shifts (Table 3, Figure 2). Points which had to be omitted correspond to reactions whose CEF values do not show correlation with the change in expression levels of the genes encoding the enzymes catalyzing these reactions. It is highly probable that these fluxes are regulated at the post-transcriptional and/or the metabolic level. The qualitative agreement between CEF and mRNA changes for up-regulation and down-regulation (points in first and third quadrants) was above 76% for all cases. These results (Table 3) indicate that fluxes through the central carbon metabolism reactions are mainly transcriptionally regulated in carbon shift experiments. However, there was no correlation in the oxygen shift experiment (Table 3, Figure 3), for which 19 data points had to be discarded to reach the threshold correlation (R^2 ^= 0.60). Plots of mRNA ratios versus CEF ratios for all cases studied are shown in Figure 2. Our results also revealed that central metabolic genes are predominantly upregulated in response to a shift from fermentative carbon source to a carbon source consisting of C-2 or C-3 compounds as most of the points lie in the first quadrant in Figure 2.

## Discussion

The correlation between mRNA ratios of the genes and corresponding CEF ratios were investigated for the genes belonging to central carbon metabolism and amino acid metabolism separately using the M81 model.

### Correlation between mRNA ratios for the genes of central carbon metabolism and corresponding CEF ratios

Our results indicate that the change in fluxes in the central carbon metabolism is to a large extent controlled at the transcriptional level for carbon source perturbations (Table 3). However, the same genes are found to be weakly correlated with CEFs in the case of oxygen shift, indicating that the response of the same genes to different perturbations is not necessarily shaped by a similar control mechanism.

For each carbon source perturbation, a small set of genes whose mRNA ratios were weakly correlated with the CEF ratios were omitted; e.g. 3 genes, *pfk12*, *fbp1 *and *pyc12*, which displayed a weak correlation as a response to a diauxic shift in batch cultures [20] (Table 3, Figure 2). Two of these genes (*fbp1*, *pfk12*) are responsible for the expression of enzymes involved in conversion between fructose-6-phosphate and fructose-diphosphate in reverse directions.*fbp1 *is known to be active during growth on ethanol whereas *pfk12 *is active during growth on glucose. Although their up or down regulation matches well with CEF predictions, there is no quantitative correlation. In other words, relatively insensitive responses at the level of gene expression may indicate an amplified transmission of the signal after the transcriptional level. However, investigation of another dataset [21] for the same respiro-fermentative shift shows better correlation for these genes and corresponding CEF values as shown in Figure 2a by the square points. Therefore, these genes may also be false-negatives resulting from the absence of replicates in the dataset.

For the glucose/ethanol shift in the chemostat culture, the glycolysis pathway genes (*pfk12*,*pyc12*, *ald45*,*pda12-pdb1*,*fba1*/*tpi1/tdh1*) were to be omitted from the analysis to reach R^2 ^0.60, indicating that these genes undergo other kinds of regulation (Table 3). This is supported by a recent study, which shows that glycolytic genes are regulated at the proteomic level in response to the same perturbation [22]. Our results indicate a good correlation between the magnitude of change in CEFs and transcript levels of genes, with the exception of these six data points. Here, we get better correlation than the comparison made using MFA based fluxes from [3], where 19 out of 43 genes could not be included in the quantitative correlation analysis since the corresponding MFA-based fold change was either zero or infinity, and the fold changes of 21 of the remaining points showed a correlation above the threshold (R^2 ^= 0.60), with a slope several folds higher than unity (3.5). This indicates that the use of CEF approach which reflects different metabolic capabilities of the microorganism for growth on a given carbon source (as reflected in the EFMs) results in a better representation of the hierarchical control, i.e. control at the transcriptional level, on metabolic fluxes, compared with focusing on a single flux distribution predicted by MFA. This is also valid for the diauxic shift in batch cultures, where our CEF approach gives a 82% qualitative agreement (Table 3), which is superior to the FBA approach that only gives 61 % qualitative agreement [6] which is based on the number of up-regulated and down-regulated points that are in agreement between experimental and simulation results.

For the glucose/acetate shift in chemostat cultures, 3 of the 6 omitted genes belong to pentose phosphate pathway (*rki1*, *sol34*, *zwf1*). The other three are from different pathways, *lsc12 *from TCA cycle, *fba1 *and *adh145 *from glycolytic pathway. Unlike chemostat cultures, lack of transcriptional regulation through fluxes of reactions governed by two different genes, namely *idp23 *and *mae1*, is implied in the case of batch cultures for a glucose-acetate shift (Table 3). However, they are closely linked since both sets are directly involved in NADPH metabolism. Analysis of an additional dataset [23] with limited data points (27) for batch cultures resulted in a very high correlation without any omission (R^2 ^= 0.78, y = 0.92 ×) (figure not shown).

For genes of the central carbon metabolism predictions by M81 was better than that of M57, meaning that further incorporation of the possible paths spanning amino acid pathways reflects the flexibility of the metabolic network better (results not shown).

### Correlation between mRNA ratios for the genes of amino acid pathways and corresponding CEF ratios

For respiratory chemostat datasets, it is difficult to establish a correlation between the ratios of expression levels of amino acid genes and corresponding CEF ratios since numerical values of both experimental mRNA and model CEF ratios for these genes are very close to unity. Therefore, these genes do not have pronounced effect on the resultant overall correlation and slope.

For respiro-fermentative batch datasets, on average ten more data points had to be removed from the graph to get the predetermined correlation of R^2 ^0.60. That is, there was a lack of correlation between the ratios of expression levels of amino acid pathway genes and corresponding CEF ratios. The observed lack of correlation for amino acid genes was also found for *E. coli *[11]. There was no positive correlation between 5 genes available in the model belonging to amino acid metabolism, consistent with what we find here. However, it should be noted that as we only looked at carbon source perturbations there were only small changes in the fluxes towards amino acids, and hence these results may not be suitable to dissect whether there is transcriptional control of fluxes towards amino acid biosynthesis. Additionally, the lack of correlation in batch cultures may be explained by the use of rich media in these fermentations. Due to the availability of amino acids in the medium, the amino acids may not have been synthesized within the cell, and this may be another cause of the poor correlation found here. Further analysis of the amino acid biosynthetic pathways therefore requires specially designed experiments.

In another study where we compared the significance of statistical change in both transcriptome and metabolome profiles of *S. cerevisiae *under different growth conditions [24], we concluded that almost all of the genes governing amino acid metabolism were metabolically regulated with or without transcriptional regulation. Although we cannot state that our current study supports this finding due to the reasons mentioned above, still it seems that there is less transcriptional regulation of metabolic fluxes in the amino acid biosynthesis compared with the central carbon metabolism. We have therefore focused our further discussion on the central carbon metabolism.

### Effect of strains and media on the transcriptional regulation of fluxes

The fluxes of the central carbon metabolism are found to be mainly transcriptionally regulated in response to the carbon source perturbations studied here (Table 3). In order to investigate the effect of strain type on the regulation of fluxes, the experimental datasets for glucose-acetate shifts in batch cultures [25] was used. That study looked at identification of changes in the transcriptome as a response to the same type of perturbation for two different yeast strains (W303 and SK1). The result presented in Table 3 is for the W303 strain and indicates that the fluxes of the central carbon metabolism of this strain is subject to transcriptional regulation, with only 3 genes being outliers. The analysis of the other strain (SK1) revealed a requirement for omission of 7 additional genes in order to reach the threshold correlation coefficient (R^2 ^= 0.60). This result suggests that the regulation behaviour can strongly depend on the genotype of the strain itself as suggested elsewhere [24,26-29].

The W303 strain is suggested to exhibit more fermentative behaviour than the SK1 strain in a glucose containing medium [25]. The expression levels of genes involved in respiratory metabolism were higher for the SK1 strain than for the W303 strain. This information was used to test our approach of distinguishing active EFMs operating in respiratory and repiro-fermentative growth. CEF analysis and comparison of CEF and mRNA ratios for SK1 were performed considering all EFMs for glucose growth instead of taking only those co-producing biomass with any by-products, assuming that those producing only biomass must also be active in this strain displaying a more respiratory behaviour. This led to a requirement for omission of 8 genes for the SK1 strain, instead of 10. On the other hand, use of all EFMs for the W303 strain caused an increase in the number of required omissions to 5. This shows that incorporation of information on the phenotypic/fermentative behaviour of the strain into the analysis may improve the prediction of the fluxes that are transcriptionally regulated.

In order to further investigate the sensitivity of our approach, we analyzed data from a study on the influence of the transcriptional response to a glucose-lactate shift [30]. That study analyzed the transcriptome during a glucose-lactate shift in carbon source with both a YPD and a complete synthetic medium. The results in Table 3 are for the YPD medium and a similar analysis was performed for the data on the complete synthetic medium. The number of the omitted fluxes for the complete synthetic medium was found to be 8, indicating that there is an effect of medium components on the regulation type of particular fluxes. Thus, we find that our method is somewhat sensitive to the actual conditions. However, still this analysis supports our overall finding that there is a fairly strong correlation between CEFs and transcriptional responses upon shifts in carbon sources.

## Conclusion

In the present study, a methodology was presented to investigate the hierarchical transmission of transcriptome changes to flux level using control effective fluxes. The high degree of correlation between the transcriptome and the fluxome obtained by the CEF approach shows that the major reason for lack of correlation reported so far was due to neglecting the flexibility of the network in operation. The detailed analysis using CEFs calculated based on the active EFMs in a particular growth type showed that fluxes in the central carbon metabolism are predominantly regulated at the transcriptional level in response to changes in carbon source. Regulation of amino acid metabolism seems to be mainly at the metabolic level; however, a definite conclusion can not be drawn since the analyzed perturbations were not directly related to this metabolism. This leads us to the hypothesis that if an applied perturbation has a direct effect on a metabolic pathway, then the genetic response of that pathway at the mRNA level is propagated into the fluxome, as demonstrated for the central carbon metabolism in this study.

## Methods

### Formulation

EFMs were calculated using FluxAnalyzer 5.3 [31]. CEF calculations were performed under a MATLAB 7.0 environment, and they were based on the efficiencies of calculated EFMs in terms of the chosen cellular objectives: production of biomass itself and ATP for maintenance. Efficiency of an EFM was determined by dividing the flux corresponding to the cellular objective by the sum of all fluxes through reactions of that mode. *j *is the index for EFMs, and *i *is the index for fluxes.

εj,CELLOBJ=rCELLOBJj∑i|rij|
 MathType@MTEF@5@5@+=feaafiart1ev1aaatCvAUfKttLearuWrP9MDH5MBPbIqV92AaeXatLxBI9gBaebbnrfifHhDYfgasaacH8akY=wiFfYdH8Gipec8Eeeu0xXdbba9frFj0=OqFfea0dXdd9vqai=hGuQ8kuc9pgc9s8qqaq=dirpe0xb9q8qiLsFr0=vr0=vr0dc8meaabaqaciaacaGaaeqabaqabeGadaaakeaaiiGacqWF1oqzdaWgaaWcbaGaemOAaOMaeiilaWIaem4qamKaemyrauKaemitaWKaemitaWKaem4ta8KaemOqaiKaemOsaOeabeaakiabg2da9maalaaabaGaemOCai3aa0baaSqaaiabdoeadjabdweafjabdYeamjabdYeamjabd+eapjabdkeacjabdQeakbqaaiabdQgaQbaaaOqaamaaqafabaWaaqWaaeaacqWGYbGCdaqhaaWcbaGaemyAaKgabaGaemOAaOgaaaGccaGLhWUaayjcSdaaleaacqWGPbqAaeqaniabggHiLdaaaaaa@4F4A@

Thereby, EFMs which are equivalent in terms of cellular objectives were distinguished by assuming that the shorter EFMs were more efficient as reflected in the denominator of the formulation [11,17]. This approach coincides with the recently suggested flux minimization objective [32], which implies that the optimum flux distribution is the one which has the minimum total flux.

Control-effective flux (*v*_*i*_) of a particular reaction (rij
 MathType@MTEF@5@5@+=feaafiart1ev1aaatCvAUfKttLearuWrP9MDH5MBPbIqV92AaeXatLxBI9gBaebbnrfifHhDYfgasaacH8akY=wiFfYdH8Gipec8Eeeu0xXdbba9frFj0=OqFfea0dXdd9vqai=hGuQ8kuc9pgc9s8qqaq=dirpe0xb9q8qiLsFr0=vr0=vr0dc8meaabaqaciaacaGaaeqabaqabeGadaaakeaacqWGYbGCdaqhaaWcbaGaemyAaKgabaGaemOAaOgaaaaa@30FE@) was determined by weighting that flux through all EFMs with the efficiencies of the corresponding modes.

vi=∑CELLOBJ1rCELLOBJmax⁡∑jεj,CELLOBJ|rij|∑jεj,CELLOBJ
 MathType@MTEF@5@5@+=feaafiart1ev1aaatCvAUfKttLearuWrP9MDH5MBPbIqV92AaeXatLxBI9gBaebbnrfifHhDYfgasaacH8akY=wiFfYdH8Gipec8Eeeu0xXdbba9frFj0=OqFfea0dXdd9vqai=hGuQ8kuc9pgc9s8qqaq=dirpe0xb9q8qiLsFr0=vr0=vr0dc8meaabaqaciaacaGaaeqabaqabeGadaaakeaacqWG2bGDdaWgaaWcbaGaemyAaKgabeaakiabg2da9maaqafabaWaaSaaaeaacqaIXaqmaeaacqWGYbGCdaqhaaWcbaGaem4qamKaemyrauKaemitaWKaemitaWKaem4ta8KaemOqaiKaemOsaOeabaGagiyBa0MaeiyyaeMaeiiEaGhaaaaaaeaacqWGdbWqcqWGfbqrcqWGmbatcqWGmbatcqWGpbWtcqWGcbGqcqWGkbGsaeqaniabggHiLdGcdaWcaaqaamaaqafabaacciGae8xTdu2aaSbaaSqaaiabdQgaQjabcYcaSiabdoeadjabdweafjabdYeamjabdYeamjabd+eapjabdkeacjabdQeakbqabaGcdaabdaqaaiabdkhaYnaaDaaaleaacqWGPbqAaeaacqWGQbGAaaaakiaawEa7caGLiWoaaSqaaiabdQgaQbqab0GaeyyeIuoaaOqaamaaqafabaGae8xTdu2aaSbaaSqaaiabdQgaQjabcYcaSiabdoeadjabdweafjabdYeamjabdYeamjabd+eapjabdkeacjabdQeakbqabaaabaGaemOAaOgabeqdcqGHris5aaaaaaa@6F27@

Here we used the ratio of CEFs of reactions at two different conditions to correlate with the expression ratios of metabolic genes responsible for the enzymes of the reactions in *S. cerevisiae*.

### Methodology

The methodology is depicted in Figure 1. Logarithms of the CEF and mRNA ratios for reactions/genes between two conditions were plotted against each other. A genetic algorithm approach was used to identify data points which caused the largest deviation from a pre-selected linear correlation (R^2^) of 0.60 ensuring as well that the slope was between 0.80–1.25, and these points were omitted from the plot. The script written in MATLAB first identifies maximum possible regression coefficient value (R^2^) for an allowed number of simultaneous omissions. And, then, the number of omissions required to reach cut-off value (0.60) is determined. The correlation coefficient value of 0.60 was selected as the threshold for an acceptable degree of correlation since it corresponds to a Pearson correlation coefficient around 0.80, which is considered to be the lower limit for a good correlation [33-35]. Moreover, the correlation between logarithmic mRNA ratios of two different wild type strains [25] in response to the same carbon shift was around 0.70 with the slope being noticeably different from unity. This inherent variability in cell behaviour depending on its genotype cannot be reflected into metabolic stoichiometry since stoichiometric models are not strain-specific, which also justifies the selected threshold value.

The number of data point omissions required to keep the regression coefficient, R^2^, above 0.60 was assumed to be one of the criteria for identifying the type of regulation imposed on the fluxes for a particular carbon shift. If many points are to be omitted to reach the threshold, this means that: a) fluxes are not transcriptionally regulated, but regulated at the translational or post-translational level (i.e. other hierarchical control mechanisms are active) or b) there is predominant metabolic regulation corresponding to substantial changes in the metabolite levels, or c) there is a combination of these two types of regulation. In addition, a second qualitative criterion, which is based on the number of points in the first (up-regulation) and third (down-regulation) quadrants of the plotted coordinate axis, was also used, as employed by others [6].

Alternatively, a statistical outlier analysis for the regressions can be employed by calculating confidence intervals on the residuals at a confidence level (e.g. 0.05), and thereby identifying outliers as those points whose residual confidence intervals do not contain zero. In this study, we preferred to use a single cut-off value for all cases analyzed. We think that this is reasonable since the correlations for all the cases was highly significant (p-value below ~10^-8^) even when no points were omitted from the dataset. Additionally, the selected cut-off value (R^2 ^= 0.60) ensures that the retained dataset after omitting points to reach the cut-off is free of outliers in all cases (data not shown). In other words, the chosen cut-off is above all R^2 ^values determined by our statistical outlier analysis. One disadvantage with statistical analysis is that it does not allow us to constrain the slope (between 0.80 and 1.25) in addition to constraining the correlation coefficient. Since statistical outlier analysis leads to the omission of fewer points than in the original analysis, it supports our conclusions.

## Authors' contributions

TÇ, BK, KÖÜ and JN designed the research. TÇ performed computational work. The manuscript was written by TÇ, BK, ZİÖ, KÖÜ and JN. All authors read and approved the final version.

## Supplementary Material

Additional file 1 Metabolic reaction set of *S. cerevisiae* Click here for file

## Figures and Tables

**Table 1:** Transcriptome datasets used in this study.

Article	Source change	Fermentation type
DeRisi et al., 1997	Carbon: Glucose- Ethanol	Batch
Lapujade et al., 2004	Carbon: Glucose-Ethanol	Chemostat
Williams et al., 2002	Carbon: Glucose-Acetate	Batch
Lapujade et al., 2004	Carbon: Glucose-Acetate	Chemostat
Prokisch et al., 2004	Carbon: Glucose-Lactate	Batch
Piper et al., 2002	Oxygen: Aerobic-Anaerobic	Chemostat

**Table 2:** Number of EFMs for each studied carbon source and with the biomass composition reported in [16].

Substrate	EFMs – M81*	EFMs – M57
Glucose	136,925 (184,631)	13,255
Ethanol	11,427 (15,099)	1,225
Acetate	4,240 (5,452)	536
Lactate	25,484 (34,319)	2,533

The numbers in paranthesis shows EFM numbers when the biomass composition of [19] is employed for comparison. EFM numbers for a smaller model (M57) are also given for comparison. The larger model (M81) includes 81 reactions and contains part of the amino acid biosynthesis together with the central carbon metabolism. The smaller model only covers the central metabolism with 57 reactions. *In M81, the EFMs with simultaneous occurrence of GDH2 and GDH13 were not taken into account since this leads to transhdrogenase activity, which is known to be not available in *S. cerevisiae*.

**Table 3:** Results of simulations for genes belonging to central carbon metabolism. Corresponding figures are given in figure 2 and 3.

		*Qualitative*	*Quantitative*				
	Actual EFM number used in simulations	Qualitative agreement*	Omissions for R^2^:0.60	Slope	Correlation coefficient (R^2^)	Omitted Genes	Not Applicable Genes^#^

Glucose/Ethanol, batch	127,872/11,427	0.82 (36/44)	3^$^	1.06	0.65	*PFK12*^$^*FBP1*^$^*PYC12*	*(GPP1-HOR2)*
Glucose/Ethanol, chemostat	9,600/7,051	0.77 (33/43)	6	0.81	0.60	*PFK12 PYC12 ALD45 (PDA12-PDB1) FBA1*^§^*TPI1*^§^, *TDH1*^§^	*(GPP1-HOR2) BPH1*
Glucose/Acetate, batch	127,872/4,238	0.76 (31/41)	3	1.11	0.63	*MAE1 IDP23 RPE1*	*(GPP1-HOR2) BPH1 PYC12 PFK12*
Glucose/Acetate, chemostat	9,600/4,190	0.78 (32/41)	6	1.18	0.61	*ADH145 RKI1 LSC12 SOL34 FBA1 ZWF1*	*(GPP1-HOR2) BPH1 PYC12 PFK12*
Glucose/Lactate, batch	127,872/25,482	0.84 (38/45)	4	0.89	0.60	*PFK12 IDP23 PYK12 MAE1*	*-*
Aerobic/Anaerobic, chemostat	9,600/8,363	0.80 (33/41)	19	1.23	0.60		

*Data points in first/third quadrants of the plots. The points with a fold change between 0.95–1.05 for either of model or experiment were considered to be in qualitative agreement.

^$^These genes were found to exhibit better agreement with CEF ratios in the analysis of [21].

^#^CEF ratio was either zero or infinity for these genes. Therefore, they could not be used in the correlation calculation.

^§ ^Omission of any two of these there points additional to the other four reported points is enough to get a correlation above the cut-off value, resulting in the same slope.
