# Supplementary material for: Effect of carbon source perturbations on transcriptional regulation of metabolic fluxes in Saccharomyces cerevisiae
Source: BMC Syst Biol. 2007 Mar 27;1:18. doi: 10.1186/1752-0509-1-18 (PMC1855933; doi:10.1186/1752-0509-1-18)
Supplement: Additional file 1 — Metabolic reaction set of S. cerevisiae [file 1752-0509-1-18-S1.pdf]

ADDITIONAL FILE 1: METABOLIC REACTION SET OF *S. CEREVISIAE*

The reaction network contains 55 central metabolic reactions including the secretion & uptake mechanisms (governed by 91 genes), and 25 reactions belonging to the amino acid metabolism (governed by 46 genes). There is one biomass formation reaction additionally. External metabolites were written in bold font in their uptake and secretion reactions. For the simulation of M81 model, reactions 1-79, 80b, and 81b was used. For M57 model, reactions 1-55, 80a and 81a was used.

	**1. Substrate Uptake**	
(1a)	1 **GLUC** + 1 ATP $\rightarrow$ 1 GLUC6P + ADP	*GLK1, HXK1,2*
(1b)	1 **ETOH** + 1 NAD_{cyt} $\rightarrow$ 1 ACAL + 1 $NADH_{cyt}$	*ADH2*
(1c)	$\rightarrow$ 1 AC	
(1d)	1 ATP + 1 **GOH** $\rightarrow$ 1 ADP + 1 GOH3P	
(1e)	1 NADcyt + 1 **LAC** $\rightarrow$ 1 NADHcyt + 1 PYR	
(2)	1 $\mathbf{NH_3}$ $\rightarrow$	*MEP1,2,3*
	**2. Glycolysis &Gluconeogenesis**[a]	
(3)	1 GLUC6P $\leftrightarrow$ 1 FRUC6P	*PGI1*
(4)	1 FRUC6P + 1 ATP $\rightarrow$ 1 FRUCDP + ADP	*PFK1,2*
(5)	1 FRUCDP $\rightarrow$ 1 FRUC6P	*FBP1*
(6)	1 FRUCDP $\leftrightarrow$ 1 GA3P + 1 DHAP	*FBA1*
(7)	1 DHAP $\leftrightarrow$ 1 GA3P	*TPI1*
(8)	1 GA3P + 1 NAD_{cyt} $\leftrightarrow$ 1 P13G + 1 $NADH_{cyt}$	*TDH1,2,3*
(9)	1 P13G + 1 ADP $\leftrightarrow$ 1 P3G + 1 ATP	*PGK1*
(10)	1 P3G $\leftrightarrow$ 1 P2G	*GPM1,2,3*
(11)	1 P2G $\leftrightarrow$ 1 PEP	*ENO1,2, ERR1,2,3*
(12)	1 PEP + 1 ADP $\rightarrow$ 1 PYR + 1 ATP	*PYK1,2*
(13)	1 DHAP + 1 $NADH_{cyt}$ $\rightarrow$ 1 GOH3P + 1 NAD_{cyt}	*GPD1,2*
(14)	1 GOH3P $\rightarrow$ 1 GOH	*GPP1, HOR2*
(15)	1 PYR $\rightarrow$ 1 ACAL + 1 CO_2	*PDC1,5,6*
(16)	1 ACAL + 1 $NADH_{cyt}$ $\leftrightarrow$ 1 ETOH + 1 NAD_{cyt}	*ADH1,4,5*
(17)	1 ACAL + 1 $NADP_{cyt}$ $\rightarrow$ 1 AC + 1 $NADPH_{cyt}$	*ALD6*
(18)	1 ACAL + 1 NAD_{mit} $\rightarrow$ 1 AC + 1 $NADH_{mit}$	*ALD4,5*
(19)	1 AC + 2 ATP $\rightarrow$ 1 $ACCOA_{cyt}$ + 2 ADP	*ACS1,2*
(20)	1 PYR + 1 NAD_{mit} $\rightarrow$ 1 $ACCOA_{mit}$ + 1 $NADH_{mit}$ + 1 CO_2	*PDA1,2, PDB1, PDX1, LPD1*

(21) 1 PYR + 1 ATP + 1 CO_2 $\rightarrow$ 1 OAC + 1 ADP *PYC1,2*

(22) 1 OAC+ 1 ATP $\rightarrow$ 1 PEP + 1 ADP + 1 CO_2 *PCK1*

3. Pentose Phosphate Pathway

(23) 1 GLUC6P + 1 $NADP_{cyt}$ $\rightarrow$ 1 G15L + 1 $NADPH_{cyt}$ *ZWF1*

(24) 1 G15L $\rightarrow$ 1 P6G *SOL3,4*

(25) 1 P6G + 1 $NADP_{cyt}$ $\rightarrow$ 1 RIBL5P + 1 $NADPH_{cyt}$ + 1 CO_2 *GND1,2*

(26) 1 RIBL5P $\leftrightarrow$ 1 RIB5P *RKI1*

(27) 1 RIBL5P $\leftrightarrow$ 1 XYL5P *RPE1*

(28) 1 RIB5P + 1 XYL5P $\leftrightarrow$ 1 SED7P + 1 GA3P *TKL1,2*

(29) 1 SED7P + 1 GA3P $\leftrightarrow$ 1 FRUC6P + 1 E4P *TAL1, YGR043C*

(30) 1 XYL5P + 1 E4P $\leftrightarrow$ 1 FRUC6P + 1 GA3P *TKL1,2*

4. Citric Acid Cycle

(31) 1 OAC + 1 $ACCOA_{mit}$ $\rightarrow$ 1 CIT *CIT1,3*

(32) 1 CIT $\leftrightarrow$ 1 ISOCIT *ACO1,2*

(33) 1 ISOCIT + 1 NAD_{mit} $\rightarrow$ 1 AKG + 1 $NADH_{mit}$ + 1 CO_2 *IDH1,2*

(34) 1 ISOCIT + 1 $NADP_{mit}$ $\rightarrow$ 1 AKG + 1 $NADPH_{mit}$ + 1 CO_2 *IDP1*

(35) 1 ISOCIT + 1 $NADP_{cyt}$ $\rightarrow$ 1 AKG + 1 $NADPH_{cyt}$ + 1 CO_2 *IDP2,3*

(36) 1 AKG + 1 NAD_{mit} $\rightarrow$ 1 SUCCOA + 1 $NADH_{mit}$ + 1 CO_2 *KGD1,2, LPD1*

(37) 1 SUCCOA + 1 ADP $\leftrightarrow$ 1 SUC + 1 ATP *LSC1,2*

(38) 1 SUC + 1 FAD $\rightarrow$ 1 FUM + 1 $FADH_2$ *SDH1,2,3,4*

(39) 1 FUM + 1 $FADH_2$ $\rightarrow$ 1 SUC + 1 FAD *OSM1*

(40) 1 FUM $\leftrightarrow$ 1 MAL *FUM1*

(41) 1 MAL + 1 NAD_{mit} $\leftrightarrow$ 1 OAC + 1 $NADH_{mit}$ *MDH1*

(42) 1 MAL + 1 $NADP_{mit}$ $\rightarrow$ 1 PYR + 1 CO_2 + 1 $NADPH_{mit}$ *MAE1*

(43) 1 $ACCOA_{cyt}$ $\rightarrow$ 1 $ACCOA_{mit}$ *CAT2, YAT1*

(44) 1 ACAL + 1 $NADH_{mit}$ $\leftrightarrow$ 1 ETOH + 1 NAD_{mit} *ADH3*

5. Glyoxylate Shunt

(45) 1 OAC + 1 $ACCOA_{cyt}$ $\rightarrow$ 1 CIT *CIT2*

(46) 1 ISOCIT $\rightarrow$ 1 GLYO + 1 SUC *ICL1,2*

(47) 1 GLYO + 1 $ACCOA_{cyt}$ $\rightarrow$ 1 MAL *MLS1,2*

(48) 1 MAL + 1 NAD_{mit} $\leftrightarrow$ 1 OAC + 1 $NADH_{cyt}$ *MDH2,3*

6. Oxidative Phosphorylation

(49) 24 ADP + 20 $NADH_{mit}$ + 10 O_2 $\rightarrow$ 24 ATP + 20 NAD_{mit} *NADHX*

(50) 24 ADP + 20 $FADH_2$ + 10 O_2 $\rightarrow$ 24 ATP + 20 FAD *FADHX*

(51) 1 ATP $\rightarrow$ 1 ADP *MAINT*

7. Product Secretion

(52)	$\rightarrow$ 1 **GOH**	*FPS1*
(53)	$\rightarrow$ 1 **ETOH**	
(54)	$\rightarrow$ 1 **AC**	*BPH1*
(55)	$\rightarrow$ 1 **SUC**	
	**8. Glutamate & Glutamine Metabolism**	
(56)	1 AKG + 1 $NADPH_{cyt}$ + 1 NH_3 $\rightarrow$ 1 GLT + 1 $NADP_{cyt}$	*GDH1,3*
(57)	1 GLT + 1 NAD_{cyt} $\rightarrow$ 1 AKG + 1 $NADH_{cyt}$ + 1 NH_3	*GDH2*
(58)	1 GLT + 1 ATP + 1 NH_3 $\rightarrow$ 1 GLN + 1 ADP	*GLN1*
(59)	1 AKG + 1 GLN + 1 $NADH_{cyt}$ $\rightarrow$ 2 GLT + 1 NAD_{cyt}	*GLT1*
(60)	1 GLT $\rightarrow$ 1 CO_2 + 1 GABA	*GAD1*
(61)	1 AKG + 1 GABA $\rightarrow$ 1 GLT + 1 SUCSAL	*UGA1*
(62)	1 SUCSAL + 1 $NADP_{cyt}$ $\rightarrow$ 1 SUC + 1 $NADPH_{cyt}$	*UGA2*
	**9. Aspartate & Asparagine & Alanine Metabolism**	
(63)	1 OAC + 1 GLT $\leftrightarrow$ 1 AKG + 1 ASP	*AAT1,2*
(64)	2 ATP + 1 GLN + 1 ASP $\leftrightarrow$ 2 ADP + 1 GLT + 1 ASN	*ASN1,2*
(65)	1 ASN $\rightarrow$ 1 ASP + 1 NH_3	*ASP3-4,1*
(66)	1 PYR + 1 GLT $\leftrightarrow$ 1 AKG + 1 ALA	*ALT1,2*
	**10. Leucine & Valine Metabolism**	
(67)	2 PYR $\rightarrow$ 1 CO2 + 1 ACLAC	*ILV2,6*
(68)	1 ACLAC + 1 $NADPH_{mit}$ $\rightarrow$ 1 $NADP_{mit}$ + 1 DHVAL	*ILV5*
(69)	1 DHVAL $\rightarrow$ 1 OIVAL	*ILV3*
(70)	$ACCOA_{mit}$ + 1 OIVAL $\rightarrow$ 1 IPPMAL	*LEU4*
(71)	1 IPPMAL + 1 NAD_{cyt} $\rightarrow$ 1 $NADH_{cyt}$ + 1 OICAP + 1 CO_2	*LEU2*
(72)	1 OICAP + 1 GLT $\leftrightarrow$ 1 AKG + 1 LEU	*BAT1,2*
(73)	1 OIVAL + 1 GLT $\leftrightarrow$ 1 AKG + 1 VAL	*BAT2*
	**11. Serine & Glycine Metabolism**	
(74)	1 P3G + NAD_{cyt} $\rightarrow$ 1 PHP + 1 $NADH_{cyt}$	*SER3,33*
(75)	1 PHP + 1 GLT $\rightarrow$ 1 AKG + 1 P3SER	*SER1*
(76)	1 P3SER $\rightarrow$ 1 SER	*SER2*
(77)	1 SER $\leftrightarrow$ 1 GLY + 1 C1	*SHM1,2*
(78)	1 ALA + 1 GLYO $\leftrightarrow$ 1 PYR + 1 GLY	*AGX1*
(79)	1 GLY + NAD_{mit} $\rightarrow$ 1 C1 + $NADH_{mit}$ + CO_2 + NH_3	*GCV1*
	**12. AICAR synthesis**	
(80a)	1 R5P + 1 P3G + 8 ATP + 1 NAD_{mit} + 1 NAD_{cyt} + 2 $NADPH_{cyt}$ $\rightarrow$ 1 $NADH_{mit}$ + 1 $NADH_{cyt}$ + 2 $NADP_{cyt}$ + 8 ADP + 1 AICAR	*ADE4,5,7,8,6,5, 2,1,13- CCM*
(80b)	1 R5P + 6 ATP + 2 GLN + 1 GLY + 1 C1 + 1 CO_2 + ASP $\rightarrow$ 2 GLU + 1 FUM	*ADE4,5,7,8,6,5,*

+ 6 ADP + 1 AICAR *2,1,13 -CCMAA*

13. Biomass Formation

(81a) 0.05877 PYR + 0.03293 OAC + 0.03430 AKG + 0.01767 P3G + 0.04302 $ACCOA_{cyt}$ + 0.00996 $ACCOA_{mit}$ + 0.00802 RB5P + 0.00889 E4P + 0.01777 PEP + 0.04400 G6P + 0.00187 GOH3P + 0.00201 AICAR + 0.28658 $NADPH_{cyt}$ + 0.03080 $NADPH_{mit}$ + 0.05056 NAD_{cyt} + 0.01716 NAD_{mit} + 1.80302 ATP *BIOMX-CCM (1 C-mol)*

$\rightarrow$

0.05056 $NADH_{cyt}$ + 0.01716 $NADH_{mit}$ + 0.28658 $NADP_{cyt}$ + 0.03080 $NADP_{mit}$ + 1.80302 ADP + 0.05806 CO2 + 1 BIOM

(81b) 0.00555 PYR + 0.04302 $ACCOA_{cyt}$ + 0 $ACCOA_{mit}$ + 0.00802 RB5P + 0.00889 E4P + 0.01777 PEP + 0.04400 G6P + 0.00187 GOH3P + 0.04485GLT + 0.02116 GLN + 0.01545 ALA + 0.03703 ASP + 0.00343 ASN + 0.00885 SER + 0.00976 GLY + 0.00996 ILE + 0.00892 VAL + 0.00595 C1 + 0.00201 AICAR + 0.12717 $NADPH_{cyt}$ + 0.01192 $NADPH_{mit}$ + 0.02198 NAD_{cyt} + 0.01716 NAD_{mit} + 1.77156 ATP *BIOMX-CCM-AA (1 C-mol)*

$\rightarrow$

0.02198 $NADH_{cyt}$ + 0.01716 $NADH_{mit}$ + 0.12717 $NADP_{cyt}$ + 0.01192 $NADP_{mit}$ + 1.77156 ADP + 0.02922 CO_2 + 1 **BIOM** + 0.00754 OAC + 0.03171 AKG + 0.00094 P3G + 0.00650 NH_3

[a]Reversible reactions were written in the direction of their occurrence in glycolysis.
